# The Predictive Value of Clinical and Molecular Characteristics or Immunotherapy in Non-Small Cell Lung Cancer: A Meta-Analysis of Randomized Controlled Trials

**DOI:** 10.3389/fonc.2021.732214

**Published:** 2021-09-07

**Authors:** Yangyang Xu, Qin Wang, Jingyuan Xie, Mo Chen, Hongbing Liu, Ping Zhan, Tangfeng Lv, Yong Song

**Affiliations:** ^1^Department of Respiration, First People’s Hospital of Changzhou, Third Affiliated Hospital of Soochow University, Changzhou, China; ^2^Department of Respiratory and Critical Care Medicine, Jinling Hospital, Nanjing Medical University, Nanjing, China; ^3^Department of Respiratory and Critical Care Medicine, Jinling Hospital, Nanjing University School of Medicine, Nanjing, China

**Keywords:** immune checkpoint inhibitor, non-small cell lung cancer, efficacy, predictor, meta-analysis

## Abstract

**Background:**

This meta-analysis aimed to investigate the efficacy of immune checkpoint inhibitor (ICI)-based therapy in non-small cell lung cancer (NSCLC) patients with different clinical and molecular characteristics such as age, sex, histological type, performance status (PS), smoking status, driver mutations, metastatic site, region and number of prior systemic regimens.

**Methods:**

A systematic literature search was conducted in PubMed, Embase, and the Cochrane library databases to identify qualified randomized controlled trials (RCTs). The primary endpoint was overall survival (OS), and the secondary endpoint was progression-free survival (PFS).

**Results:**

A total of 19 RCTs were included in this meta-analysis. ICI-based therapy significantly improved OS compared with non-ICI therapy in patients aged <65 years (HR, 0.74; P<0.00001), 65-74 years (HR, 0.73; P<0.00001), receiving first-line (HR, 0.75; P<0.00001) or second-line (HR, 0.72; P<0.00001) treatment, current or previous smokers (HR, 0.76; P<0.00001), and EGFR wild-type patients (HR, 0.76; P<0.00001), but not in patients aged ≥75 years (HR, 0.91; P=0.50), receiving third-line treatment (HR, 0.93; P=0.55), never smokers (HR, 0.84; P=0.10), or EGFR mutant patients (HR, 0.99; P=0.92). No statistical OS improvement was observed in KRAS mutant (HR, 0.68; P=0.05) or KRAS wild-type (HR, 0.95; P=0.65) patients. Immunotherapy improved OS in NSCLC patients, regardless of sex (male or female), histological type (squamous or non-squamous NSCLC), PS (0 or 1), metastatic site (brain or liver metastases), and region (East Asia or America/Europe) (all P<0.05). Subgroup analysis showed that the survival benefit of ICIs in patients with brain metastases was observed in first-line combination therapy (P<0.05), but not in second or more line monotherapy (P>0.05). Programmed death-1 (PD-1) inhibitors significantly prolonged OS in patients with liver metastases compared with non-ICI therapy (P=0.0007), but PD-L1 inhibitors did not (P=0.35). Similar results were observed in the combined analysis of PFS.

**Conclusions:**

Age, smoking status, EGFR mutation status, and number of prior systemic regimens predicted the efficacy of immunotherapy. While sex, histological type, PS 0 or 1, KRAS mutation status and region were not associated with the efficacy of ICIs. Patients with liver metastases benefited from anti-PD-1-based therapy, and those with brain metastases benefited from first-line ICI-based combination therapy.

**Systematic Review Registration:**

http://www.crd.york.ac.uk/prospero, identifier CRD42020206062.

## Introduction

Immunotherapy is a key and effective method for the treatment of cancer patients, which improves the treatment mode of cancer. Immune checkpoint inhibitors (ICIs) can block cytotoxic T lymphocyte antigen-4 (CTLA-4) or programmed death-1 (PD-1) pathway and inhibit the release of negative regulatory factors of immune activation to enhance anti-tumor response (1). To date, a number of large-scale randomized controlled trials (RCTs) have demonstrated that ICIs represented by programmed death-1 (PD-1)/programmed death-ligand 1 (PD-L1) inhibitors, whether used as monotherapy or as combination therapy, provide long-term survival and lasting benefits for patients with non-small cell lung cancer (NSCLC) (2–6).

However, the survival benefits are observed in only a small number of patients (15%-25%), and the majority of patients have primary or acquired resistance to ICIs. Considering the high cost of immunotherapy and immune-related adverse reactions, it is necessary to explore appropriate biomarkers to find patients suitable for immunotherapy and to achieve accurate treatment of lung cancer (7). Our previous meta-analysis has demonstrated that PD-L1 expression detected by immunohistochemical is an effective biomarker for predicting the efficacy of checkpoint inhibitors in NSCLC. Patients with high levels of PD-L1 expression are more likely to benefit from anti-PD-1/PD-L1 therapy (8). However, the detection of PD-L1 expression depends on the patient’s tissue sample, which is difficult to obtain and the sample size is usually very small. Moreover, in practical application, there are various antibody clones and assays, which provide challenges for the detection of PD-L1 expression (9). Tumor mutation burden (TMB) is another predictive biomarker of widespread concern. Whether TMB can clearly predict the efficacy of immunotherapy remains controversial. The KEYNOTE-158 study prospectively explored the relationship between high tissue TMB and the efficacy of pembrolizumab (anti-PD-1 antibody), and found that patients with high TMB had better response rates (10). In the exploratory analyses of KEYNOTE-021 and KEYNOTE-189, there was no significant correlation between TMB and the efficacy of immunotherapy (11, 12). Therefore, it is of great value to explore other economic and practical factors to predict the efficacy of immunotherapy. In some prespecified subgroups of RCTs, the effects of immunotherapy varied among patients with different clinical and molecular characteristics such as age, sex, race, Eastern Cooperative Oncology Group (ECOG) performance status (PS) score, and so on. For example, in IMpower 130, there was no significant difference in overall survival (OS) between the atezolizumab plus chemotherapy group and the chemotherapy group among male, patients aged <65 years and ≥65 years, current or previous smokers, never smokers, or with liver metastases (2). In the CheckMate 017 trial, nivolumab significantly improved survival in male, patients aged <75 years, and in the region of US/Canada or Europe, but not in female, patients aged ≥75 years, and in the rest-of-world region (4). Checkmate 227 found that no survival improvement of ICIs was observed in female, patients aged 65-74 years, ≥75 years, ECOG PS 1, never smokers, non-squamous NSCLC, with liver metastases or brain metastases (5). Thus, a pooled analysis of relevant RCTs is needed to further investigate whether clinical or molecular factors can predict survival in NSCLC patients receiving immunotherapy.

In this meta-analysis, we conducted a systematic review to comprehensively compare the efficacy of anti-PD-1/PD-L1-based therapy and non-ICI therapy in patients with different >clinical and molecular characteristics, and to identify people who are more likely to benefit from immunotherapy. We present the following article in accordance with the PRISMA reporting checklist.

## Materials and Methods

### Search Strategy

The review was registered in PROSPERO before the start of this study (ID: CRD42020206062). Two authors independently conducted a systematic literature search in PubMed, Embase, and the Cochrane library databases, and the deadline for the search was July 15, 2020. The following keywords were included in our search: (“immunotherapy” or “PD-1” or “PD-L1” or “nivolumab” or “pembrolizumab” or “atezolizumab” or “durvalumab” or “avelumab”) AND (“lung cancer” or “lung neoplasms” or “lung carcinoma” or “NSCLC”). When necessary, the references cited in published articles were searched manually.

### Study Selection and Data Extraction

The inclusion criteria designed according to PICOS structure were as follows: (I) Population: NSCLC patients. (II) Intervention: ICI group (including doublet ICIs, PD-1/PD-L1 inhibitors used alone or in combination with chemotherapy +/- angiogenesis inhibitors). (III) Control: non-ICI group (including chemotherapy +/- angiogenesis inhibitors). (IV) Outcomes: OS or progression-free survival (PFS) of prespecified subgroups by age, sex, region, ECOG PS score, smoking status, brain metastases, liver metastases, driver mutations, histological type and number of prior systemic regimens. (V) Study: RCTs. (VI) All studies were available in full text. Studies in which survival data were insufficient or the control group received only placebo were excluded. If more than one study reported the same trial, we included the latest study with the largest number of patients and the longest follow-up. If several articles reported different subgroups of the same trial, we included them all.

Two authors independently extracted the following data from the included studies: name of the first author, year of publication, name of the RCT, trial phase, study population, line of therapy, treatment regimen, number of patients, and survival outcomes of the prespecified subgroups. Any inconsistencies were resolved through consultation.

### Quality Assessment and Statistical Analysis

Two authors independently assessed the risk of bias of the included studies by Cochrane Bias tool. Any inconsistencies were resolved by consensus. The primary endpoint of the study was to compare OS between the ICI group and the non-ICI group, which was measured by the hazard ratio (HR) and the corresponding 95% confidence interval (CI). The secondary endpoint was PFS. If HRs or the corresponding 95%CI were not directly reported in the text, we extracted them manually by plotting on the forest plot with a logarithmic scale. In addition, considering the possible sources of heterogeneity, we predesigned the following three subgroup analyses to compare OS between the two groups: line of therapy, treatment regimen, and target of ICIs. The heterogeneity was tested by Cochrane Q test and I^2^ values. P <0.1 or I^2^ >50% was considered to have significant heterogeneity, and the random effect model was used; otherwise, the fixed effect model was used. Potential publication bias was evaluated by funnel plot. We performed sensitivity analysis by excluding trials with small sample size or excluding studies in which HR and the corresponding 95%CI could not be obtained directly. RevMan software (Review Manager, Version 5.4.1, The Cochrane Collaboration, 2020) was used for all statistical analysis, and P value <0.05 was considered statistically significant.

## Results

### Study Selection and Characteristics

**Figure 1:**
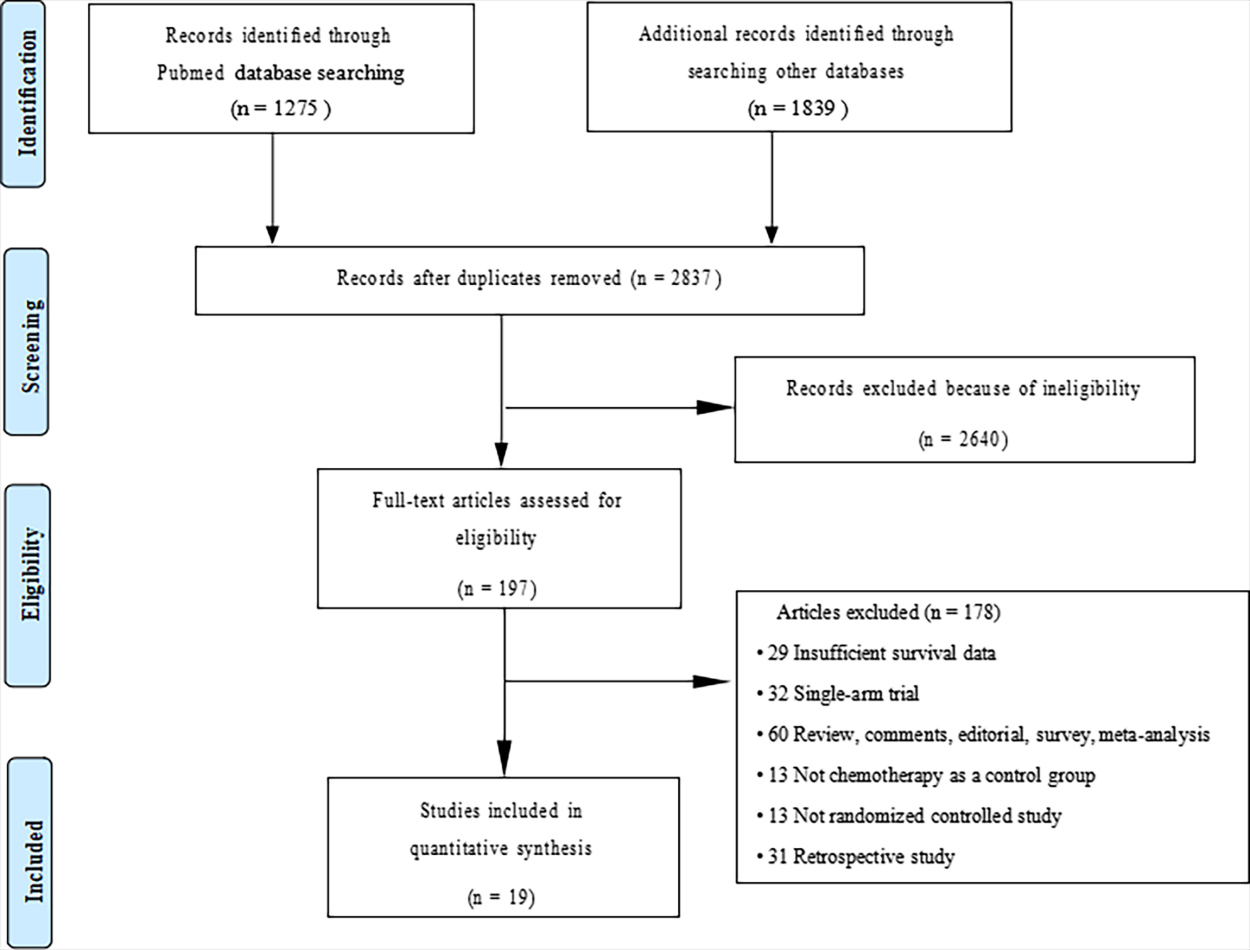
Flowchart of study selection.

**Figure 2:**
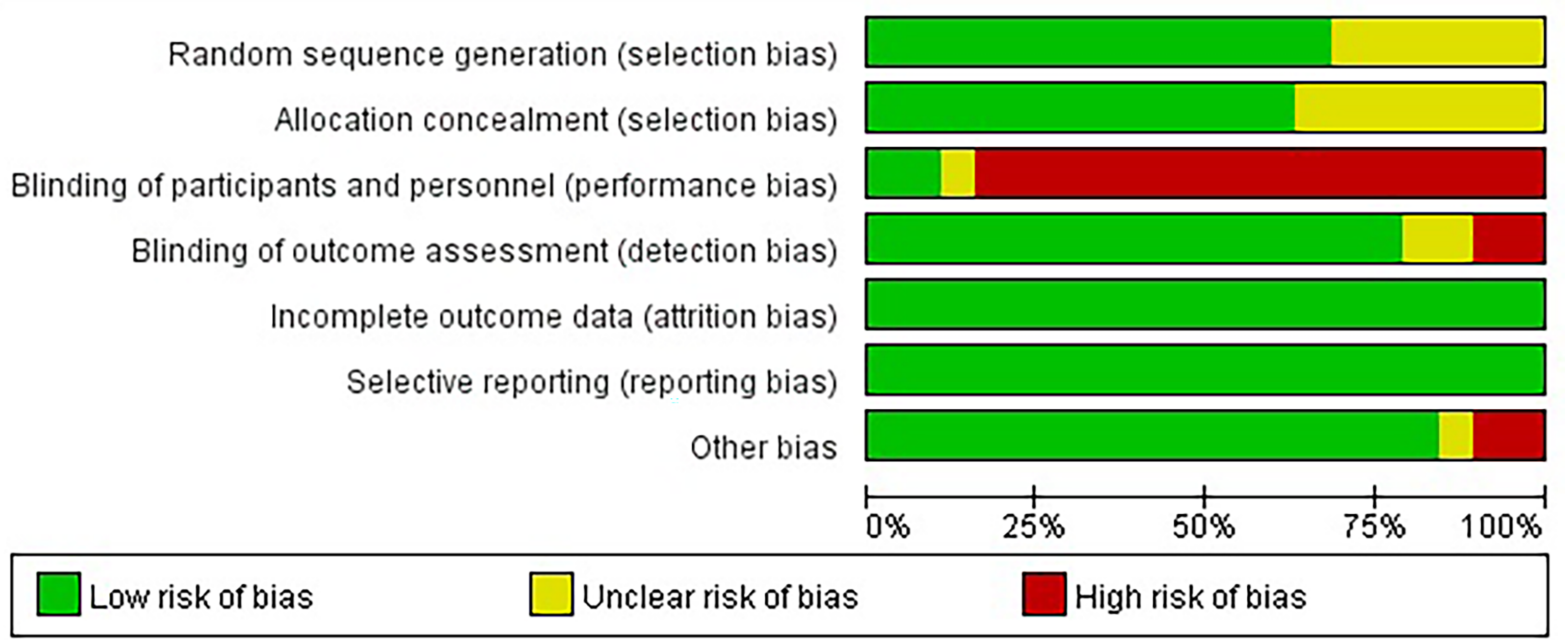
Risk of bias graph.

We initially screened a total of 3114 articles, of which 277 were excluded due to duplication. According to the pre-defined inclusion and exclusion criteria, a total of 19 RCTs involving 11983 patients were eventually included. **Figure 1** shows a flowchart of the selection process for the study. Among the included trials, three studies were phase II trials (3, 13, 14), one was phase II/III trial (15, 16), and fifteen were phase III trials (2, 4–6, 17–31). IMpower150 study included two experimental groups: carboplatin plus paclitaxel plus atezolizumab, and carboplatin plus paclitaxel plus bevacizumab plus atezolizumab, all of which were compared with the control group: carboplatin plus paclitaxel plus bevacizumab (18, 19). Notably, although KEYNOTE-407 released updated efficacy results in 2020, there was no eligible subgroup analysis data and therefore it was not included in our meta-analysis (32). The baseline characteristics of the included studies are shown in **Table 1**. Detailed results of the risk of bias for each study are shown in **Figure 2**. Overall, all RCTs had low risk of bias.

**Table 1:** Study characteristics of included randomized controlled trials.

Source	Trial	Phase	Study population	Line of therapy	Stratification criteria	Experimental arm (N)	Control arm (N)	Primary endpoint
Fehrenbacher, 2016	POPLAR	II	NSCLC	≥2L	Histology, smoking status	Atezolizumab (144)	Docetaxel (143)	OS
Jotte, 2020	IMpower131	III	Stage IV squamous NSCLC	1L	Age, sex, race, ECOG PS, smoking status, liver metastases, histology, number of prior systemic regimens	Atezolizumab+carboplatin+nab-paclitaxel (343)	Carboplatin+nab-paclitaxel (340)	PFS and OS
West, 2019	IMpower130	III	Stage IV non-squamous NSCLC, no EGFR/ALK mutations	1L	Age, sex, ECOG PS, smoking status, liver metastases, histology, number of prior systemic regimens	Atezolizumab+carboplatin+nab-paclitaxel (451)	Carboplatin+nab-paclitaxel (228)	PFS and OS
Reck, 2019/Socinski, 2018	IMpower150	III	Stage IV or recurrent metastatic non-squamous NSCLC	1L	Age, sex, EGFR, KRAS, liver metastases, histology, number of prior systemic regimens	Arm A: Atezolizumab+carboplatin+paclitaxel (402); Arm B: Atezolizumab+carboplatin+paclitaxel+bevacizumab (400)	Carboplatin+paclitaxel+bevacizumab (400)	PFS and OS
Barlesi, 2018	JAVELIN Lung 200	III	Stage IIIB/IV or recurrent NSCLC, PD-L1 ≥ 1%	≥2L	Age, sex, ECOG PS, histology, smoking status, region	Avelumab (264)	Docetaxel (265)	OS
Rizvi, 2020	MYSTIC	III	Stage IV NSCLC without sensitizing EGFR/ALK mutations, PD-L1 ≥ 25%	1L	Age, sex, histology, smoking status, race, ECOG PS	Durvalumab (163)	Chemotherapy (platinum-based doublet chemotherapy) (162)	OS
Borghaei, 2015/Vokes, 2018	CheckMate 057	III	Stage IIIB/IV or recurrent non-squamous NSCLC	≥2L	Age, number of prior systemic regimens, sex, ECOG PS, smoking status, region, EGFR, KRAS, brain metastases, liver metastases, histology	Nivolumab (292)	Docetaxel (290)	OS
Brahmer, 2015/Vokes, 2018	CheckMate 017	III	Stage IIIB or IV squamous NSCLC	2L	Age, sex, region, ECOG PS, smoking status, liver metastases, histology, number of prior systemic regimens	Nivolumab (135)	Docetaxel (137)	OS
Carbone, 2017	CheckMate 026	III	Stage IV or recurrent NSCLC, PD-L1 ≥ 1%	1L	Age, sex, ECOG PS, histology, smoking status, number of prior systemic regimens	Nivolumab (271)	Chemotherapy (platinum doublet chemotherapy) (270)	PFS
Wu, 2019	CheckMate 078	III	Stage IIIB/IV or recurrent NSCLC, no EGFR/ALK mutations	≥2L	Age, sex, ECOG PS, smoking status, brain metastases, histology, region	Nivolumab (338)	Docetaxel (166)	OS
Mok, 2019	KEYNOTE-042	III	Locally advanced or metastatic NSCLC without a sensitising EGFR/ALK mutation,PD-L1 ≥ 1%	1L	Age, sex, ECOG PS, histology, smoking status, number of prior systemic regimens, region	Pembrolizumab (637)	Chemotherapy (platinum-based chemotherapy) (637)	OS
Arrieta, 2020	PROLUNG	II	Advanced NSCLC	≥2L	EGFR	Pembrolizumab+docetaxel (40)	Docetaxel (38)	ORR
Hellmann, 2019	CheckMate 227	III	Stage IV or recurrent NSCLC	1L	Age, sex, ECOG PS, smoking status, histology, liver metastases, brain metastases, number of prior systemic regimens	Nivolumab+ipilimumab (583)	Chemotherapy (583)	OS
Paz-Ares, 2018	KEYNOTE-407	III	Metastatic, stage IV squamous NSCLC	1L	Age, sex, ECOG PS, histology, number of prior systemic regimens, region	Pembrolizumab+carboplatin+paclitaxel/nab-paclitaxel (278)	Placebo+carboplatin+paclitaxel/nab-paclitaxel (281)	PFS and OS
Gandhi, 2018/Gadgeel, 2020	KEYNOTE-189	III	Metastatic non-squamous NSCLC, no EGFR/ALK mutations	1L	Age, sex, ECOG PS, smoking status, brain metastases, liver metastases, histology, number of prior systemic regimens	Pembrolizumab+pemetrexed+platinum (410)	Placebo+pemetrexed+platinum (206)	PFS and OS
Herbst, 2016/Herbst, 2020	KEYNOTE-010	II/III	NSCLC with PD-L1 ≥ 1%	≥2L	Age, sex, ECOG PS, histology, EGFR	The pooled pembrolizumab doses (690): pembrolizumab (2 mg/kg every 3 weeks) (344)/pembrolizumab (10 mg/kg every 3 weeks) (346)	Docetaxel (343)	PFS and OS
Reck, 2016/Reck, 2019	KEYNOTE-024	III	Stage IV NSCLC without EGFR/ALK mutations, PD-L1 ≥ 50%	1L	Age, sex, ECOG PS, histology, smoking status, brain metastases, number of prior systemic regimens, region	Pembrolizumab (154)	Chemotherapy (platinum-based chemotherapy) (151)	PFS
Fehrenbacher, 2018	OAK	III	NSCLC	≥2L	Age, sex, histology, ECOG PS, number of prior systemic regimens, smoking status, brain metastases, EGFR, KRAS, histology, region	Atezolizumab (613)	Docetaxel (612)	OS
Borghaei, 2018	KEYNOTE-021	II	Stage IIIB/IV non-squamous NSCLC, no EGFR/ALK mutations	1L	Histology, number of prior systemic regimens	Pembrolizumab+chemotherapy (60)	Chemotherapy (pemetrexed+carboplatin) (63)	ORR

NSCLC, Non-small cell lung cancer; N, Number; 1L, First line; ≥2L, ≥ Second line; PD-L1, Programmed death-ligand 1; ECOG PS, Eastern Cooperative Oncology Group performance status; EGFR, Epidermal growth factor receptor; ALK, Anaplastic lymphoma kinase; KRAS, Kirsten RAS; OS, Overall survival; PFS, Progression-free survival; ORR, Objective response rate.

### Effects of ICIs by Age

**Figure 3:**
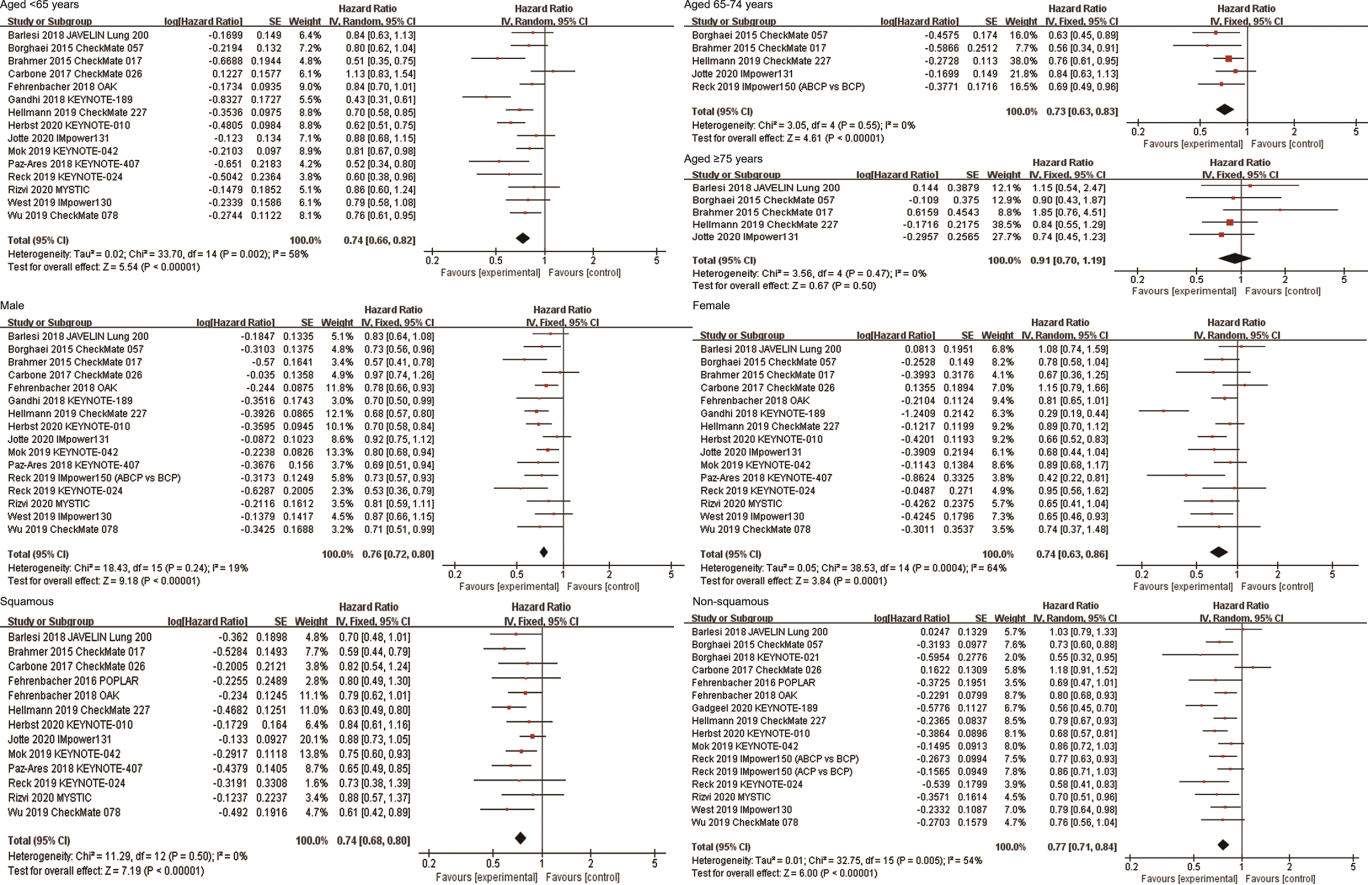
Forest plots of hazard ratios comparing overall survival between patients treated with anti-PD-1/PD-L1-based therapy or non-ICI therapy according to age, sex, and histological type. PD-1, programmed death-1; PD-L1, programmed death-ligand 1; ICI, immune checkpoint inhibitor.

A total of 15 studies reported OS data of NSCLC patients stratified by age, including 15 studies for patients aged <65 years, 5 studies for patients aged 65-74 years, and 5 studies for patients aged ≥75 years. Among patients aged <65 years (HR, 0.74; 95%CI, 0.66-0.82; P<0.00001) and aged 65-74 years (HR, 0.73; 95%CI, 0.63-0.83; P<0.00001), immunotherapy significantly improved OS compared with non-ICI treatment. However, among patients aged ≥75 years, there was no significant difference in survival between the two groups (HR, 0.91; 95%CI, 0.70-1.19; P=0.50, **Figure 3**). Subgroup analyses based on the line of therapy, treatment regimen, and target of ICIs showed that these factors did not affect the OS improvement of ICIs in patients aged <65 years and 65-74 years. However, no prolonged survival was observed in patients aged ≥75 years, regardless of the line of therapy, treatment regimen, and target of ICIs (**Table 2**). In terms of PFS, the combined HR for patients aged <65 years, 65-74 years and ≥75 years were 0.72 (95%CI, 0.62-0.84; P<0.0001), 0.66 (95%CI, 0.50-0.86; P=0.003) and 0.80 (95%CI, 0.60-1.06; P=0.12), respectively (**Supplementary Figure 1**).

**Table 2:** Subgroup analysis comparing OS in patients with different clinical and molecular characteristics.

Population	Subgroup	No. of studies	Test of association			Test of heterogeneity	
			HR	95% CI	P value	I²	P value
Aged <65 years	Total	15	0.74	0.66-0.82	<0.00001	58%	0.002
	Line of therapy						
	1L	9	0.74	0.62-0.87	0.0004	66%	0.003
	≥2L	6	0.73	0.64-0.84	<0.0001	50%	0.08
	Treatment regimen						
	monotherapy	10	0.77	0.69-0.87	<0.0001	51%	0.03
	combination therapy	5	0.66	0.52-0.84	0.0006	69%	0.01
	Target of ICIs						
	PD-1	10	0.68	0.59-0.79	<0.00001	67%	0.001
	PD-L1	5	0.84	0.75-0.95	0.004	0%	0.99
Aged 65-74 years	Total	5	0.73	0.63-0.83	<0.00001	0%	0.55
	Line of therapy						
	1L	3	0.77	0.66-0.90	0.0009	0%	0.66
	≥2L	2	0.61	0.46-0.80	0.0005	0%	0.67
	Treatment regimen						
	monotherapy	2	0.61	0.46-0.80	0.0005	0%	0.67
	combination therapy	3	0.77	0.66-0.90	0.0009	0%	0.66
	Target of ICIs						
	PD-1	3	0.70	0.59-0.83	<0.0001	0%	0.42
	PD-L1	2	0.77	0.62-0.96	0.02	0%	0.36
Aged ≥75 years	Total	5	0.91	0.70-1.19	0.50	0%	0.47
	Line of therapy						
	1L	2	0.80	0.58-1.11	0.18	0%	0.71
	≥2L	3	1.19	0.75-1.87	0.46	0%	0.47
	Treatment regimen						
	monotherapy	3	1.19	0.75-1.87	0.46	0%	0.47
	combination therapy	2	0.80	0.58-1.11	0.18	0%	0.71
	Target of ICIs						
	PD-1	3	0.99	0.66-1.48	0.95	19%	0.29
	PD-L1	2	0.85	0.56-1.29	0.45	0%	0.34
Male	Total	16	0.76	0.72-0.80	<0.00001	19%	0.24
	Line of therapy						
	1L	10	0.78	0.72-0.84	<0.00001	32%	0.15
	≥2L	6	0.73	0.67-0.81	<0.00001	0%	0.51
	Treatment regimen						
	monotherapy	10	0.76	0.70-0.82	<0.00001	22%	0.24
	combination therapy	6	0.76	0.69-0.84	<0.00001	27%	0.23
	Target of ICIs						
	PD-1	10	0.72	0.67-0.78	<0.00001	21%	0.25
	PD-L1	6	0.82	0.75-0.90	<0.0001	0%	0.77
Female	Total	15	0.74	0.63-0.86	0.0001	64%	0.0004
	Line of therapy						
	1L	9	0.70	0.54-0.90	0.006	76%	<0.0001
	≥2L	6	0.77	0.68-0.88	0.0001	5%	0.38
	Treatment regimen						
	monotherapy	10	0.82	0.73-0.92	0.001	17%	0.29
	combination therapy	5	0.56	0.37-0.85	0.006	82%	0.0001
	Target of ICIs						
	PD-1	10	0.72	0.57-0.90	0.004	73%	0.0001
	PD-L1	5	0.78	0.65-0.92	0.004	19%	0.30
Squamous	Total	13	0.74	0.68-0.80	<0.00001	0%	0.50
	Line of therapy						
	1L	7	0.76	0.68-0.84	<0.00001	10%	0.35
	≥2L	6	0.72	0.63-0.82	<0.00001	0%	0.52
	Treatment regimen						
	monotherapy	10	0.74	0.67-0.82	<0.00001	0%	0.81
	combination therapy	3	0.72	0.57-0.91	0.005	67%	0.05
	Target of ICIs						
	PD-1	8	0.69	0.62-0.76	<0.00001	0%	0.66
	PD-L1	5	0.83	0.73-0.94	0.003	0%	0.84
Non-squamous	Total	16	0.77	0.71-0.84	<0.00001	54%	0.005
	Line of therapy						
	1L	10	0.77	0.68-0.87	<0.0001	64%	0.003
	≥2L	6	0.77	0.69-0.86	<0.00001	32%	0.19
	Treatment regimen						
	monotherapy	10	0.79	0.71-0.89	<0.0001	58%	0.01
	combination therapy	6	0.74	0.65-0.84	<0.00001	52%	0.06
	Target of ICIs						
	PD-1	9	0.74	0.65-0.85	<0.0001	69%	0.001
	PD-L1	7	0.81	0.75-0.88	<0.00001	0%	0.49
ECOG PS 0	Total	15	0.75	0.68-0.82	<0.00001	22%	0.21
	Line of therapy						
	1L	9	0.74	0.62-0.88	0.0008	46%	0.06
	≥2L	6	0.75	0.65-0.87	<0.0001	0%	0.68
	Treatment regimen						
	monotherapy	10	0.77	0.68-0.86	<0.00001	0%	0.45
	combination therapy	5	0.70	0.55-0.90	0.006	55%	0.07
	Target of ICIs						
	PD-1	10	0.72	0.64-0.82	<0.00001	29%	0.18
	PD-L1	5	0.79	0.68-0.92	0.002	11%	0.34
ECOG PS 1	Total	12	0.72	0.66-0.79	<0.00001	50%	0.02
	Line of therapy						
	1L	7	0.73	0.65-0.82	<0.00001	40%	0.12
	≥2L	5	0.72	0.60-0.85	0.0002	66%	0.02
	Treatment regimen						
	monotherapy	7	0.72	0.63-0.83	<0.00001	63%	0.01
	combination therapy	5	0.72	0.64-0.83	<0.00001	31%	0.22
	Target of ICIs						
	PD-1	8	0.67	0.60-0.76	<0.00001	49%	0.06
	PD-L1	4	0.81	0.73-0.91	0.0003	4%	0.37
Current or previous smoker	Total	14	0.76	0.70-0.82	<0.00001	44%	0.04
	Line of therapy						
	1L	8	0.77	0.68-0.89	0.0002	63%	0.009
	≥2L	6	0.74	0.68-0.81	<0.00001	0%	0.58
	Treatment regimen						
	monotherapy	10	0.77	0.70-0.85	<0.00001	37%	0.11
	combination therapy	4	0.74	0.62-0.88	0.0005	65%	0.03
	Target of ICIs						
	PD-1	8	0.72	0.62-0.83	<0.00001	63%	0.008
	PD-L1	6	0.81	0.74-0.88	<0.00001	0%	0.94
Never smoker	Total	13	0.84	0.68-1.03	0.10	43%	0.05
	Line of therapy						
	1L	8	0.76	0.57-1.02	0.07	46%	0.07
	≥2L	5	0.94	0.69-1.29	0.69	46%	0.11
	Treatment regimen						
	monotherapy	9	0.93	0.77-1.13	0.48	14%	0.32
	combination therapy	4	0.61	0.34-1.10	0.10	69%	0.02
	Target of ICIs						
	PD-1	7	0.83	0.62-1.11	0.20	50%	0.06
	PD-L1	6	0.84	0.60-1.18	0.32	46%	0.10
EGFR mutant	Total	5	0.99	0.76-1.28	0.92	0%	0.61
	Line of therapy						
	1L	2	0.79	0.49-1.25	0.31	0%	0.39
	≥2L	3	1.09	0.80-1.48	0.58	0%	0.72
	Treatment regimen						
	monotherapy	3	1.09	0.80-1.48	0.58	0%	0.72
	combination therapy	2	0.79	0.49-1.25	0.31	0%	0.39
	Target of ICIs						
	PD-1	2	1.04	0.70-1.53	0.86	0%	0.50
	PD-L1	3	0.95	0.68-1.34	0.78	7%	0.34
EGFR wildtype	Total	3	0.72	0.65-0.79	<0.00001	0%	0.58
	Target of ICIs						
	PD-1	2	0.68	0.60-0.78	<0.00001	0%	0.78
	PD-L1	1	0.76	0.65-0.89	0.0006	–	–
KRAS mutant	Total	2	0.68	0.46-0.99	0.05	17%	0.27
KRAS wildtype	Total	2	0.95	0.75-1.20	0.65	0%	0.79
Brain metastases	Total	6	0.64	0.52-0.80	<0.0001	30%	0.21
	Line of therapy						
	1L	3	0.54	0.39-0.74	0.0001	5%	0.35
	≥2L	3	0.76	0.56-1.04	0.08	26%	0.26
	Treatment regimen						
	monotherapy	4	0.76	0.56-1.02	0.07	0%	0.44
	combination therapy	2	0.53	0.38-0.73	0.0001	47%	0.17
	Target of ICIs						
	PD-1	5	0.66	0.51-0.85	0.001	43%	0.14
	PD-L1	1	0.59	0.38-0.92	0.02	–	–
Liver metastasis	Total	7	0.78	0.68-0.90	0.0007	39%	0.13
	Line of therapy						
	1L	6	0.82	0.70-0.96	0.01	42%	0.12
	≥2L	1	0.67	0.50-0.91	0.01	–	–
	Treatment regimen						
	monotherapy	1	0.67	0.50-0.91	0.01	–	–
	combination therapy	6	0.82	0.70-0.96	0.01	42%	0.12
	Target of ICIs						
	PD-1	3	0.73	0.60-0.87	0.0007	0%	0.49
	PD-L1	4	0.85	0.61-1.19	0.35	57%	0.07
East Asia	Total	5	0.72	0.60-0.86	0.0002	16%	0.31
	Line of therapy						
	1L	3	0.69	0.54-0.90	0.005	49%	0.14
	≥2L	2	0.74	0.58-0.94	0.01	0%	0.40
	Treatment regimen						
	monotherapy	4	0.74	0.62-0.89	0.001	0%	0.42
	combination therapy	1	0.44	0.22-0.89	0.02	–	–
	Target of ICIs						
	PD-1	4	0.69	0.56-0.84	0.0002	24%	0.27
	PD-L1	1	0.84	0.57-1.25	0.40	–	–
America/Europe	Total	4	0.71	0.57-0.88	0.002	57%	0.07
	Target of ICIs						
	PD-1	2	0.56	0.44-0.72	<0.00001	0%	0.43
	PD-L1	2	0.80	0.70-0.91	0.0008	0%	0.44
0 prior therapy	Total	11	0.75	0.67-0.84	<0.00001	67%	0.0007
	Treatment regimen						
	monotherapy	3	0.77	0.55-1.09	0.14	86%	0.0008
	combination therapy	8	0.74	0.67-0.82	<0.00001	50%	0.05
	Target of ICIs						
	PD-1	7	0.70	0.59-0.83	<0.0001	77%	0.0002
	PD-L1	4	0.82	0.75-0.91	<0.0001	0%	0.74
1 prior therapy	Total	3	0.72	0.65-0.81	<0.00001	32%	0.23
2 prior therapy	Total	2	0.93	0.72-1.19	0.55	41%	0.19

OS, Overall survival; No, Number; HR, hazard ratio; CI, confidence interval; 1L, First line; ≥2L, ≥ Second line; ICIs, Immune checkpoint inhibitors; PD-1, Programmed death-1; PD-L1, Programmed death-ligand 1; ECOG PS, Eastern Cooperative Oncology Group performance status; EGFR, Epidermal growth factor receptor; KRAS, Kirsten RAS.

### Effects of ICIs by Sex

Sixteen and fifteen studies respectively explored the efficacy of ICIs in male and female patients. The combined results showed that anti-PD-1/PD-L1 immunotherapy significantly improved OS of both male and female NSCLC patients compared with non-ICI treatment (HR, 0.76; 95%CI, 0.72-0.80; P<0.00001 for male; HR, 0.74; 95%CI, 0.63-0.86; P = 0.0001 for female, **Figure 3**). Subgroup analyses based on the line of therapy, treatment regimen, and target of ICIs showed that none of these factors affect the OS improvement of immunotherapy in both male and female patients (**Table 2**). In terms of PFS, the combined HRs for male and female patients were 0.69 (95%CI, 0.61-0.77; P<0.00001) and 0.82 (95%CI, 0.64-1.04; P=0.10), respectively (**Supplementary Figure 1**).

### Effects of ICIs by Histological Type

There were 13 and 16 studies on the efficacy of ICIs for squamous NSCLC and non-squamous NSCLC, respectively. The combined results showed that ICIs significantly improved OS in both squamous NSCLC (HR, 0.74; 95%CI, 0.68-0.80; P<0.00001) and non-squamous NSCLC (HR, 0.77; 95%CI, 0.71-0.84; P<0.00001, **Figure 3**). Subgroup analyses based on the line of therapy, treatment regimen, and target of ICIs showed that these factors did not affect the OS improvement of ICIs in both both squamous and non-squamous NSCLC patients (**Table 2**). In terms of PFS, the combined HRs for squamous NSCLC and non-squamous NSCLC were 0.69 (95%CI, 0.60-0.79; P<0.00001) and 0.76 (95%CI, 0.62-0.94; P=0.01), respectively (**Supplementary Figure 1**).

### Effects of ICIs by ECOG PS Score

**Figure 4:**
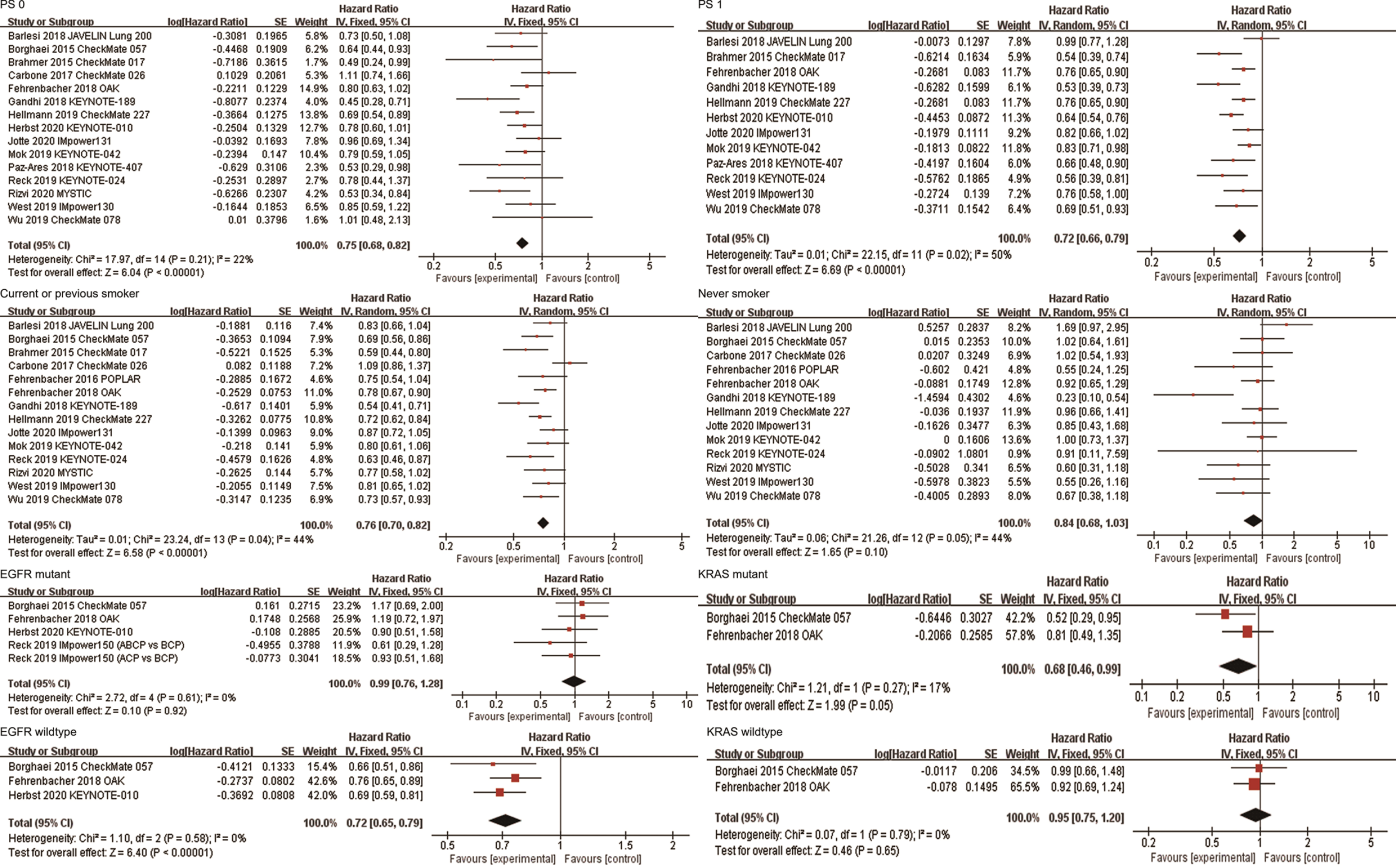
Forest plots of hazard ratios comparing overall survival between patients treated with anti-PD-1/PD-L1-based therapy or non-ICI therapy according to PS score, smoking status, and driver mutations. PD-1, programmed death-1; PD-L1, programmed death-ligand 1; ICI, immune checkpoint inhibitor; PS, performance status.

A total of 15 studies explored the efficacy of anti-PD-1/PD-L1-based therapy in patients with ECOG PS 0, and 12 studies explored the efficacy in patients with ECOG PS 1. The combined results showed that compared with non-ICI treatment, both patients with ECOG PS 0 (HR, 0.75; 95%CI, 0.68-0.82; P<0.00001) and ECOG PS 1 (HR, 0.72; 95%CI, 0.66-0.79; P<0.00001) achieved OS improvement after receiving immunotherapy (**Figure 4**). Subgroup analyses based on the line of therapy, treatment regimen, and target of ICIs showed that these factors did not affect the OS improvement of ICIs in patients with ECOG PS 0 or ECOG PS 1 (**Table 2**). In terms of PFS, the combined HRs for patients with ECOG PS 0 and ECOG PS 1 were 0.72 (95%CI, 0.57-0.91; P=0.007) and 0.68 (95%CI, 0.60-0.77; P<0.00001), respectively (**Supplementary Figure 2**).

### Effects of ICIs by Smoking Status

Fourteen studies reported the efficacy of ICIs in patients who currently or previously smoked. The combined results showed that anti-PD-1/PD-L1 therapy significantly improved OS of current or previous smokers compared with non-ICI therapy (HR, 0.76; 95%CI, 0.70-0.82; P<0.00001, **Figure 4**). Subgroup analyses based on the line of therapy, treatment regimen, and target of ICIs showed that these factors did not affect the OS improvement in current or previous smokers (**Table 2**). Thirteen studies reported the efficacy of ICIs in patients who never smoked. The combined results showed that there was no statistical difference in survival between patients receiving immunotherapy and those receiving conventional treatment (HR, 0.84; 95%CI, 0.68-1.03; P=0.10, **Figure 4**). Subgroup analysis based on the line of therapy showed that the pooled HR was 0.76 (95%CI, 0.57-1.02; P=0.07) in patients receiving first-line treatment, and 1.01 (95%CI, 0.71-1.44; P=0.95) in patients receiving second or more line treatment. Subgroup analysis by treatment regimen showed that neither immune monotherapy (HR, 0.97; 95%CI, 0.80-1.17; P=0.75) nor ICI-based combination therapy (HR, 0.61; 95%CI, 0.34-1.10; P=0.10) significantly prolonged survival in never smokers compared to non-ICI therapy. Subgroup analysis based on the target of ICIs showed that the combined HR was 0.85 (95%CI, 0.61-1.19; P=0.34) in patients receiving PD-1 inhibitors, and 0.84 (95%CI, 0.60-1.18; P=0.32) in patients receiving PD-L1 inhibitors (**Table 2**). In terms of PFS, the combined HRs for current or previous smokers and never smokers were 0.70 (95%CI, 0.60-0.81; P<0.00001) and 1.01 (95%CI, 0.70-1.44; P=0.98), respectively (**Supplementary Figure 2**).

### Effects of ICIs by Driver Mutation Status

A total of five studies reported OS data in EGFR mutation-positive patients. The pooled results showed that immunotherapy did not provide longer OS for EGFR mutation-positive patients compared with non-ICI treatment (HR, 0.99; 95%CI, 0.76-1.28; P=0.92, **Figure 4**). Subgroup analysis by the line of therapy showed that the combined HR was 0.79 (95%CI, 0.49-1.25; P=0.31) in patients receiving first-line treatment, and 1.09 (95%CI, 0.80-1.48; P=0.58) in patients receiving second-line or more treatment. Subgroup analysis by the treatment regimen showed that the combined HR was 1.09 (95%CI, 0.80-1.48; P=0.58) in patients receiving immune monotherapy, and 0.79 (95%CI, 0.49-1.25; P=0.31) in patients receiving anti-PD-1/PD-L1-based combination therapy. Subgroup analysis by the target of ICIs showed that the combined HR was 1.04 (95%CI, 0.70-1.53; P=0.86) in patients receiving PD-1 inhibitors, and 0.95 (95%CI, 0.68-1.34; P=0.78) in patients receiving PD-L1 inhibitors (**Table 2**). Three studies reported OS data for wild-type EGFR patients, all of which compared the efficacy of second-line or more immune monotherapy with docetaxel. The combined HR of patients with EGFR wild-type was 0.72(95% CI,0.65-0.79; P<0.00001, **Figure 4**). Subgroup analysis based on the target of ICIs showed that both PD-1 (HR, 0.68; 95%CI, 0.60-0.78; P<0.00001) and PD-L1 inhibitors (HR, 0.76; 95%CI, 0.65-0.89; P=0.0006) provided survival benefits for these patients. In terms of PFS, the combined HR for EGFR mutation-positive and wild-type patients were 1.00 (95%CI, 0.62-1.62; P=1.00) and 0.69 (95%CI, 0.48-0.99; P=0.04), respectively (**Supplementary Figure 2**).

Two studies (OAK and CheckMate057) reported survival outcomes in patients with different KRAS mutation status, both of which explored the efficacy of anti-PD-1/PD-L1 monotherapy in second or more line therapy. The combined HRs of KRAS mutant and wild-type patients were 0.68 (95%CI, 0.46-0.99; P=0.05) and 0.95 (95%CI, 0.75-1.20; P=0.65), respectively (**Figure 4**). In terms of PFS, the combined HRs for KRAS mutation-positive and wild-type patients were 0.64 (95%CI, 0.43-0.94; P=0.02) and 0.87 (95%CI, 0.28-2.76; P=0.82), respectively (**Supplementary Figure 2**).

### Effects of ICIs by Metastatic Sites

**Figure 5:**
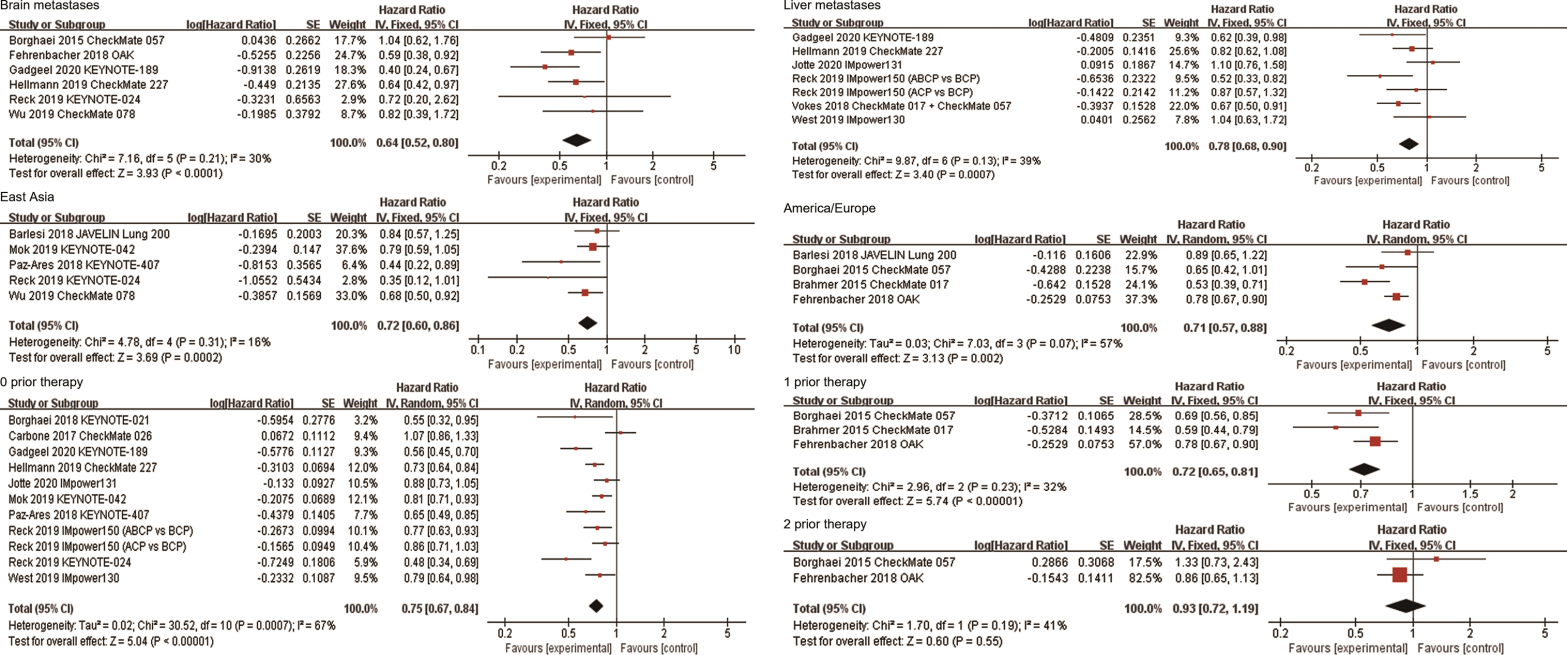
Forest plots of hazard ratios comparing overall survival between patients treated with anti-PD-1/PD-L1-based therapy or non-ICI therapy according to metastatic site, region, and number of prior systemic regimens. PD-1, programmed death-1; PD-L1, programmed death-ligand 1; ICI, immune checkpoint inhibitor.

A total of six studies reported survival data in patients with brain metastases. The combined results showed that anti-PD-1/PD-L1-based therapy was associated with longer OS in these patients (HR, 0.64; 95%CI, 0.52-0.80; P<0.0001, **Figure 5**). Subgroup analysis based on the line of therapy showed that in the first-line treatment, patients with brain metastases who received immunotherapy had better survival than those who received non-ICI treatment (HR, 0.54; 95%CI, 0.39-0.74; P=0.0001). However, there was no significant difference in survival between the two groups in second or more line therapy (HR, 0.76; 95%CI, 0.56-1.04; P=0.08). Subgroup analysis by the treatment regimen suggested that the survival benefit was observed in patients with brain metastases who received ICI-based combination therapy (HR, 0.53; 95%CI, 0.38-0.73; P=0.0001), but not in patients who received immune monotherapy (HR, 0.76; 95%CI, 0.56-1.02; P=0.07, **Table 2**). In addition, patients with brain metastasis had OS benefit regardless of the target of ICIs. The pooled HR of PFS in patients with brain metastases was 0.57 (95%CI, 0.43-0.76; P<0.0001, **Supplementary Figure 3**).

A total of 7 studies reported survival data in patients with liver metastases. Among them, Vokes et al. reported the combined survival outcomes of patients with liver metastases in CheckMate 057 and CheckMate 017 (17). The combined HR of these patients was 0.78 (95%CI, 0.68-0.90; P=0.0007, **Figure 5**). Six studies reported the efficacy of ICI-based combination therapy in the first-line treatment *versus* non-ICI treatment, among which five were PD-1/PD-L1 inhibitors combined with chemotherapy and one was PD-1 inhibitor combined with CTLA-4 inhibitor. Subgroup analysis showed that first-line ICI-based combination therapy significantly improved OS compared with chemotherapy (HR, 0.82; 95%CI, 0.70-0.96; P=0.01). In addition, the combined results of CheckMate 057 and CheckMate 017 showed that patients with liver metastases who received nivolumab in second or more line treatment had longer OS than those receiving docetaxel (HR, 0.67; 95%CI, 0.50-0.91; P=0.01). Subgroup analysis based on the target of ICIs showed that the combined HR was 0.73 (95%CI, 0.60-0.87; P=0.0007) in patients using PD-1 inhibitors, and 0.85 (95%CI, 0.61-1.19; P=0.35) in patients using PD-L1 inhibitors (**Table 2**). In addition, the pooled HR of PFS in patients with liver metastases was 0.66 (95%CI, 0.49-0.89; P=0.006, **Supplementary Figure 3**).

### Effects of ICIs by Region

Five studies reported the efficacy of immunotherapy in patients in East Asia. The combined results showed that anti-PD-1/PD-L1 therapy significantly improved OS in East Asians compared with non-ICI treatment (HR, 0.72; 95%CI, 0.60-0.86; P=0.0002, **Figure 5**). Subgroup analyses based on the line of therapy and treatment regimen showed that these factors did not affect the OS improvement of ICIs in East Asians. Subgroup analysis based on the target of ICIs showed that the combined HR of four studies on PD-1 inhibitors was 0.69 (95%CI, 0.56-0.84; P=0.0002). Only one study was related to PD-L1 inhibitor, with the HR of 0.84 (95%CI, 0.57-1.25; P=0.40, **Table 2**). Four studies reported the efficacy of ICIs in American/European patients. All of them explored the efficacy of anti-PD-1/PD-L1 monotherapy in second or more line therapy. The combined analysis showed that ICIs provided higher OS than non-ICI treatment for patients in America/Europe (HR, 0.71; 95%CI, 0.57-0.88; P=0.002, **Figure 5**). Subgroup analysis based on the target of ICIs showed that the combined HR was 0.56 (95%CI,0.44-0.72; P<0.00001) in patients receiving PD-1 inhibitors, and 0.92 (95%CI, 0.71-1.19; P=0.52) in patients receiving PD-L1 inhibitors (**Table 2**). In terms of PFS, the combined HRs for patients in East Asia and America/Europe were 0.68 (95%CI, 0.48-0.96; P=0.03) and 0.61 (95%CI, 0.40-0.95; P=0.03), respectively (**Supplementary Figure 3**).

### Effects of ICIs by Number of Prior Systemic Regimens

Eleven studies explored the efficacy of immunotherapy in patients who had not previously received systemic treatment. The combined results showed that anti-PD-1/PD-L1 therapy significantly improved OS of these patients (HR, 0.75; 95%CI, 0.67-0.84; P<0.00001, **Figure 5**). Subgroup analysis based on the treatment regimen showed that PD-1/PD-L1 inhibitor combined with other therapies significantly improved survival compared to non-ICI treatment (HR, 0.74; 95%CI, 0.67-0.82; P<0.00001), but the survival benefit was not observed in patients who received PD-1/PD-L1 inhibitor monotherapy (HR, 0.77; 95%CI, 0.55-1.09; P=0.14). Subgroup analysis by the target of ICIs showed that the combined HR was 0.70 (95%CI, 0.59-0.83; P<0.0001) in patients receiving PD-1 inhibitors, and 0.82 (95%CI, 0.75-0.91; P<0.0001) in patients receiving PD-L1 inhibitors (**Table 2**). Three studies explored the efficacy of immunotherapy in patients who had previously received one systemic treatment, all of which compared the efficacy of anti-PD-1/PD-L1 monotherapy with docetaxel in NSCLC patients. The combined HR for these patients was 0.72 (95%CI, 0.65-0.81; P<0.00001, **Figure 5**). Two studies explored the efficacy of anti-PD-1/PD-L1 monotherapy in patients who had received two prior systemic regimens, with a combined HR of 0.93 (95%CI, 0.72-1.19; P=0.55, **Figure 5**). In terms of PFS, the combined HR for patients receiving first-line, second-line and third-line treatment was 0.67 (95%CI, 0.53-0.84; P=0.0005), 0.73 (95%CI, 0.54-1.00; P=0.05), and 1.70 (95%CI, 1.00-2.90; P=0.05), respectively (**Supplementary Figure 3**).

### Sensitivity Analysis and Publication Bias

Sensitivity analysis was performed by excluding KEYNOTE-021 and PROLUNG trials because of the small number of patients included in the two studies. The results showed that the predictive value of different clinical and molecular characteristics on the OS of anti-PD-1/PD-L1-based therapy was stable. In addition, we excluded the CheckMate 078 trial, whose HR and 95%CI were estimated from forest plot, and found that the conclusions of the primary analysis did not change. Furthermore, by observing the funnel plots of OS in each subgroup, we found no obvious publication bias (**Supplementary Figures 4**–**7**).

## Discussion

Although immunotherapy has made a significant breakthrough in NSCLC, only a small number of patients benefit from ICIs. Therefore, it is of great value to explore appropriate biomarkers to guide the selection of patients suitable for immunotherapy. PD-L1 expression and TMB are currently the most widely studied biomarkers, but the detection process is complex and expensive, which brings challenges to cancer treatment. Some retrospective studies have also been conducted to try to explore novel biomarkers. Prat et al. found that PD-1 gene expression and 12 signatures tracking the activation of CD8 and CD4 T-cells, natural killer cells, and IFN were significantly associated with PFS (33). In the POPLAR trial, atezolizumab benefited survival in tumors with high expression of T-effector and IFN-γ gene signatures (3). Patients with higher ratios of central memory T cells to effector T cells had longer PFS (34). Neutrophil to lymphocyte ratio (NLR), pretreatment lactate dehydrogenase (LDH), lung immune prognostic index (based on derived NLR and LDH levels), C-reactive protein (CRP), and gut microbiome may also be potential biomarkers (35–39). However, these biomarkers have only been identified in retrospective or exploratory analyses of small samples, and their predictive value of efficacy needs to be further confirmed in prospective trials. In addition, for some biomarkers, such as NLR, LDH and CRP, there is still no uniform standard to define the relevant threshold. With the accumulation of the latest clinical data, we attempted to explore whether there are more available and cost-effective clinical and molecular pathologic factors to predict the efficacy of immunotherapy.

Our meta-analysis included 19 RCTs. We compared the survival of patients with different clinical and molecular characteristics (age, sex, histological type, ECOG PS score, smoking status, driver mutations, brain metastases, liver metastases, region and number of prior systemic regimens) who received immunotherapy with those who received non-ICI treatment. Meanwhile, we conducted pre-defined subgroup analyses according to the line of therapy, treatment regimen and target of ICIs to explore the role of ICIs in these populations. Our study found that age, EGFR mutation status, smoking status and number of prior systemic regimens could effectively predict the efficacy of immunotherapy. To the best of our knowledge, our meta-analysis is the most comprehensive study with the largest number of RCTS included, providing guidance for better identification of which patients are most likely to benefit from anti-PD-1/PD-L1 treatment.

In previous studies which investigated the relationship between age and the efficacy of immunotherapy, the cut-off age was mostly 65 years old. They found no statistical difference between the ICI group and non-ICI group in patients <65 years old and ≥65 years old (40, 41). However, it remains unclear whether elderly NSCLC patients aged ≥75 years will also benefit from immunotherapy. A multicenter retrospective study of patients aged ≥75 years found that the efficacy of ICIs in elderly patients was similar to that in young patients (42). Another study in Italy found that patients aged ≥75 years had lower median OS than patients aged <65 years or aged 65-74 years (43). Zheng et al. found that there was no significant difference in survival between the immunotherapy group and the chemotherapy group in patients older than 75 years (44). In our meta-analysis, we included 15 studies on the relationship between age and immunotherapy. Our study was the first to perform a more detailed division of age, and explore the efficacy of immunotherapy in patients aged <65 years, 65-74 years, and ≥75 years. We found that ICIs significantly improved OS compared with non-ICI treatment for patients aged <65 years and aged 65-74 years. However, in elderly patients ≥75 years old, immunotherapy did not significantly prolong the survival.

Due to the poor prognosis of NSCLC patients with distant metastasis (such as brain or liver metastases), the effect of immunotherapy on patients with different metastatic sites has been a research hotspot in recent years. Our combined analysis of six studies involving patients with asymptomatic brain metastases suggested that these patients obtained longer OS after receiving immunotherapy than non-ICI treatment. However, further subgroup analysis suggested that the survival benefit of ICIs was only observed in first-line combination therapy, but not in second or more line monotherapy. Therefore, early ICI-based combination therapy is recommended for patients with asymptomatic brain metastasis. In addition, patients with liver metastases also benefited from immunotherapy. Subgroup analysis showed that both first-line ICI-based combination therapy and second or more line anti-PD-1/PD-L1 monotherapy were associated with improved OS in patients with liver metastases. It is worth noting that PD-1 inhibitors significantly prolonged survival in patients with liver metastases compared with non-ICI therapy, while the survival benefit was not observed in patients receiving PD-L1 inhibitors. Similarly, a recent study also found that PD-1 inhibitors showed superior survival compared to PD-L1 inhibitors in cancer treatment (45). This may be because although both PD-1 inhibitors and PD-L1 inhibitors can block the binding of PD-1 to PD-L1, PD-1 inhibitors can also block the binding of PD-1 to PD-L2 (46). Previous studies suggested that PD-L2 expression was a predictor of the efficacy of ICIs independent of PD-L1 expression. Therefore, the clinical effect of immunotherapy may also be related to the blockage of the PD-1/PD-L2 pathway (46, 47).

In our meta-analysis, smoking status predicted the effect of immunotherapy. We found that survival benefits of immunotherapy were observed only in current or previous smokers, but not in never smokers. This may be because smoking is associated with high TMB, which makes it easier to benefit from ICIs (48). The number of prior systemic regimens also predicted the clinical outcome of immunotherapy. The survival benefit of ICIs was observed when the number of prior systemic regimens was 0 and 1, but it was not observed when the number of prior systemic regimens was 2. Furthermore, our study demonstrated that patients benefited from anti-PD-1/PD-L1 immunotherapy, regardless of sex (male or female), histological type (squamous or non-squamous NSCLC), ECOG PS (PS 0 or 1), and region (East Asia or America/Europe). Since most RCTs excluded patients with poor performance (PS ≥2), we did not investigate the role of ICIs in the population with PS ≥2. A recent meta-analysis, which included 19 clinical studies in real-world, found that PS ≥2 predicted worse survival in patients receiving immunotherapy (49). In the future, whether PS ≥2 is a predictor of poor immunotherapy efficacy remains to be further confirmed in RCTs. For women who received PD-1/PD-L1 inhibitors, there was a trend of PFS improvement compared with non-ICI therapy, but the difference between the two groups was not statistically significant. This may be because female have stronger immune escape mechanisms than male cancer patients, and thus are more likely to develop resistance to immunotherapy (50–52). In addition, our study indicated that women eventually achieved OS improvement, which further suggested the importance of subsequent treatment.

The relationship between driver mutation and anti-PD-1/PD-L1 therapy has been a hot topic. Our study found that EGFR mutation status was associated with the efficacy of ICIs. EGFR wild-type patients benefited from ICIs, while EGFR mutation-positive patients did not. This may be explained by the following reasons. Firstly, different from patients with wild-type EGFR, EGFR mutations influenced the anti-tumor immune response by regulating possible factors related to tumor microenvironment status (such as regulatory T cells, tumor-infiltrating lymphocytes, exosomes, etc.). Secondly, patients with EGFR sensitive mutations were more common among never-smokers, and they had significantly lower TMB than those with wild-type EGFR. Thirdly, previous studies showed that PD-L1 expression in EGFR mutant tumors was significantly lower than that in EGFR wild-type tumors, which led to poor response to anti-PD-1/PD-L1 therapy in EGFR mutant patients (53–56). Although patients with EGFR mutations generally respond poorly to ICIs, some patients may still benefit from immunotherapy. In IMpower150, EGFR mutation-sensitive patients (L858R and 19DEL) treated with atezolizumab plus bevacizumab and chemotherapy achieved an improvement in OS (18). In contrast, other retrospective studies suggested that uncommon EGFR mutations (G719X and exon 20 insertions) were positively associated with the survival benefits of immunotherapy. After disease progression during EGFR tyrosine kinase inhibitors (TKIs) treatment, patients without T790M mutations were more likely to benefit from subsequent immunotherapy (57, 58). In addition, ICI-based combination therapy (such as ICI in combination with chemotherapy or anti-angiogenic drugs) may be more effective than ICI alone in pre-treated EGFR mutant NSCLC patients (59). It has also been suggested that shorter duration of EGFR-TKI remission (<6 months) is associated with longer PFS in subsequent immunotherapy (58, 60). Smoking status may be a clinical predictor of the response to ICIs in EGFR-mutated NSCLC. An Italian study found that among patients with EGFR mutations, the median OS of current or previous smokers was higher than that of non-smokers (14.1 months *vs.* 5.6 months), although the difference was not statistically significant (P=0.12) (61). Yoshida et al. suggested that a higher Brinkman Index (≥600, defined as the number of cigarettes smoked per day multiplied by the smoking years) might be a favorable predictor for the efficacy of ICIs (58). In a word, it is currently difficult to use a single biomarker to screen potential populations of EGFR-mutated NSCLC who might benefit from immunotherapy. It is important to integrate multiple predictors to assess the outcome of immunotherapy in this population. In terms of KRAS mutation status, we did not have enough evidence to demonstrate its predictive value for the efficacy of immunotherapy. Although PFS improvement was observed in KRAS mutant patients, no statistical improvement in OS was observed in these patients. In addition, there was no statistical difference in survival between the two groups for KRAS wild-type patients. Notably, there were few studies on KRAS mutation status: 2 studies reported OS data and another 2 studies reported PFS data. In the future, it is necessary to conduct more studies to explore the relationship between KRAS mutation status and immunotherapy.

Our meta-analysis also has some limitations. First, some studies included patients with PD-L1 positive expression, which may overestimate the treatment effect of ICIs. Second, there were some differences among the included studies, such as line of therapy, treatment regimen, and target of ICIs, which may lead to heterogeneity. We used the random effect model to solve this problem and conducted subgroup analyses to explore the source of heterogeneity. At the same time, we also carried out sensitivity analyses, which confirmed the reliability of our conclusion. Third, our meta-analysis was based on the results of prespecified subgroup analyses of published RCTs, rather than studies that specifically analyzed the impact of a single clinicopathological factor on immunotherapy. There may be correlations between these clinicopathological factors, such as EGFR mutation status and its association in non-smokers. When we focus on a single feature, other confounders may influence survival outcomes.

In conclusion, age, smoking status, EGFR mutation status, and number of prior systemic regimens predicted the efficacy of immunotherapy. Patients aged <65 years or 65-74 years, receiving first-line or second-line treatment, current or previous smokers, and EGFR wild-type patients may benefit from immunotherapy. However, there was insufficient evidence to demonstrate the predictive value of KRAS mutation status for the efficacy of ICIs. PD-1/PD-L1 inhibitors improved the OS of NSCLC patients regardless of sex (male or female), histological type (squamous or non-squamous NSCLC), ECOG PS (PS 0 or 1), and region (East Asia or America/Europe). Patients with liver metastases also benefited from anti-PD-1-based therapy. In addition, first-line ICI-based combination therapy was recommended for patients with asymptomatic brain metastases. In the practical application of ICIs, the comprehensive consideration of these clinical and molecular biomarkers is helpful to better guide the treatment of NSCLC patients.

## Data Availability Statement

The original contributions presented in the study are included in the article/**Supplementary Material**. Further inquiries can be directed to the corresponding authors.

## Author Contributions 

(I) Conception and design: PZ, YS. (II) Administrative support: TL, PZ. (III) Provision of study materials or patients: JX, MC. (IV) Collection and assembly of data: HL, QW, YX. (V) Data analysis and interpretation: YX, QW. All authors contributed to the article and approved the submitted version.

## Funding

This work was supported by grants from the National Natural Science Foundation of China (grant number 81401903, 81572937 and 81572273); the 16th batch “Summit of the Six Top Talents” Program of Jiangsu Province (grant number WSN-154); China Postdoctoral Science Foundation 12th batch Special fund (postdoctoral number 45786); China Postdoctoral Science Foundation 64th batch (postdoctoral number 45786); Jiangsu Provincial Postdoctoral Science Foundation (grant number 2018K049A); the Natural Science Foundation of Jiangsu province (grant number BK20180139); Jiangsu Provincial Medical Youth Talent (grant number QNRC2016125); the Nanjing Medical Science and Technology Development Project (No. ZKX17044); and the Jiangsu Provincial Key Research and Development Program (No. BE2016721).

## Conflict of Interest

The authors declare that the research was conducted in the absence of any commercial or financial relationships that could be construed as a potential conflict of interest.

## Publisher’s Note

All claims expressed in this article are solely those of the authors and do not necessarily represent those of their affiliated organizations, or those of the publisher, the editors and the reviewers. Any product that may be evaluated in this article, or claim that may be made by its manufacturer, is not guaranteed or endorsed by the publisher.

## Supplementary Material

Supplementary Figure 1 Forest plots of hazard ratios comparing progression-free survival between patients treated with anti-PD-1/PD-L1-based therapy or non-ICI therapy according to age, sex, and histological type. PD-1, programmed death-1; PD-L1, programmed death-ligand 1; ICI, immune checkpoint inhibitor.

Supplementary Figure 2 Forest plots of hazard ratios comparing progression-free survival between patients treated with anti-PD-1/PD-L1-based therapy or non-ICI therapy according to PS score, smoking status, and driver mutations. PD-1, programmed death-1; PD-L1, programmed death-ligand 1; ICI, immune checkpoint inhibitor; PS, performance status.

Supplementary Figure 3 Forest plots of hazard ratios comparing progression-free survival between patients treated with anti-PD-1/PD-L1-based therapy or non-ICI therapy according to metastatic site, region, and number of prior systemic regimens. PD-1, programmed death-1; PD-L1, programmed death-ligand 1; ICI, immune checkpoint inhibitor.

Supplementary Figure 4 Funnel plots of overall survival in the subgroup according to age and number of prior systemic regimens.

Supplementary Figure 5 Funnel plots of overall survival in the subgroup according to sex, histological type, and PS score. PS, performance status.

Supplementary Figure 6 Funnel plots of overall survival in the subgroup according to smoking status and driver mutations.

Supplementary Figure 7 Funnel plots of overall survival in the subgroup according to metastatic site and region.

## Abbreviations

NSCLC, non-small cell lung cancer; ICI, immune checkpoint inhibitor; PS, performance status; RCTs, randomized controlled trials; OS, overall survival; PFS, progression-free survival; PD-1, programmed death-1; PD-L1, programmed death-ligand 1; CTLA-4, cytotoxic T lymphocyte antigen-4; TMB, tumor mutation burden; ECOG, Eastern Cooperative Oncology Group; CI, confidence interval.
