# Supplementary figures and images for: The Predictive Value of Clinical and Molecular Characteristics or Immunotherapy in Non-Small Cell Lung Cancer: A Meta-Analysis of Randomized Controlled Trials
Source: Front Oncol. 2021 Sep 7;11:732214. doi: 10.3389/fonc.2021.732214 (PMC8453160; doi:10.3389/fonc.2021.732214)

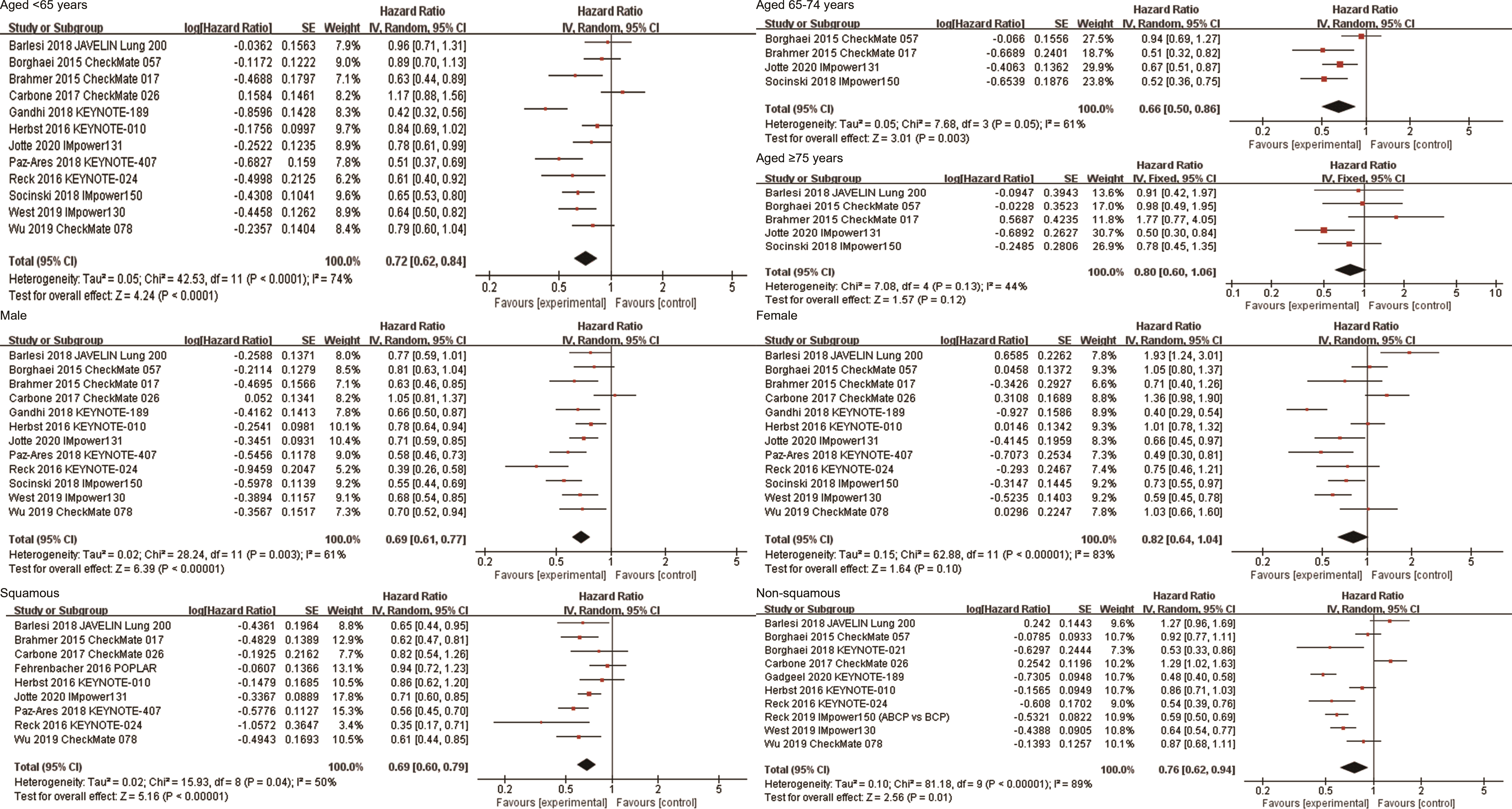

Supplement: Supplementary Figure 1 — Forest plots of hazard ratios comparing progression-free survival between patients treated with anti-PD-1/PD-L1-based therapy or non-ICI therapy according to age, sex, and histological type. PD-1, programmed death-1; PD-L1, programmed death-ligand 1; ICI, immune checkpoint inhibitor. [file Image_1.jpeg]

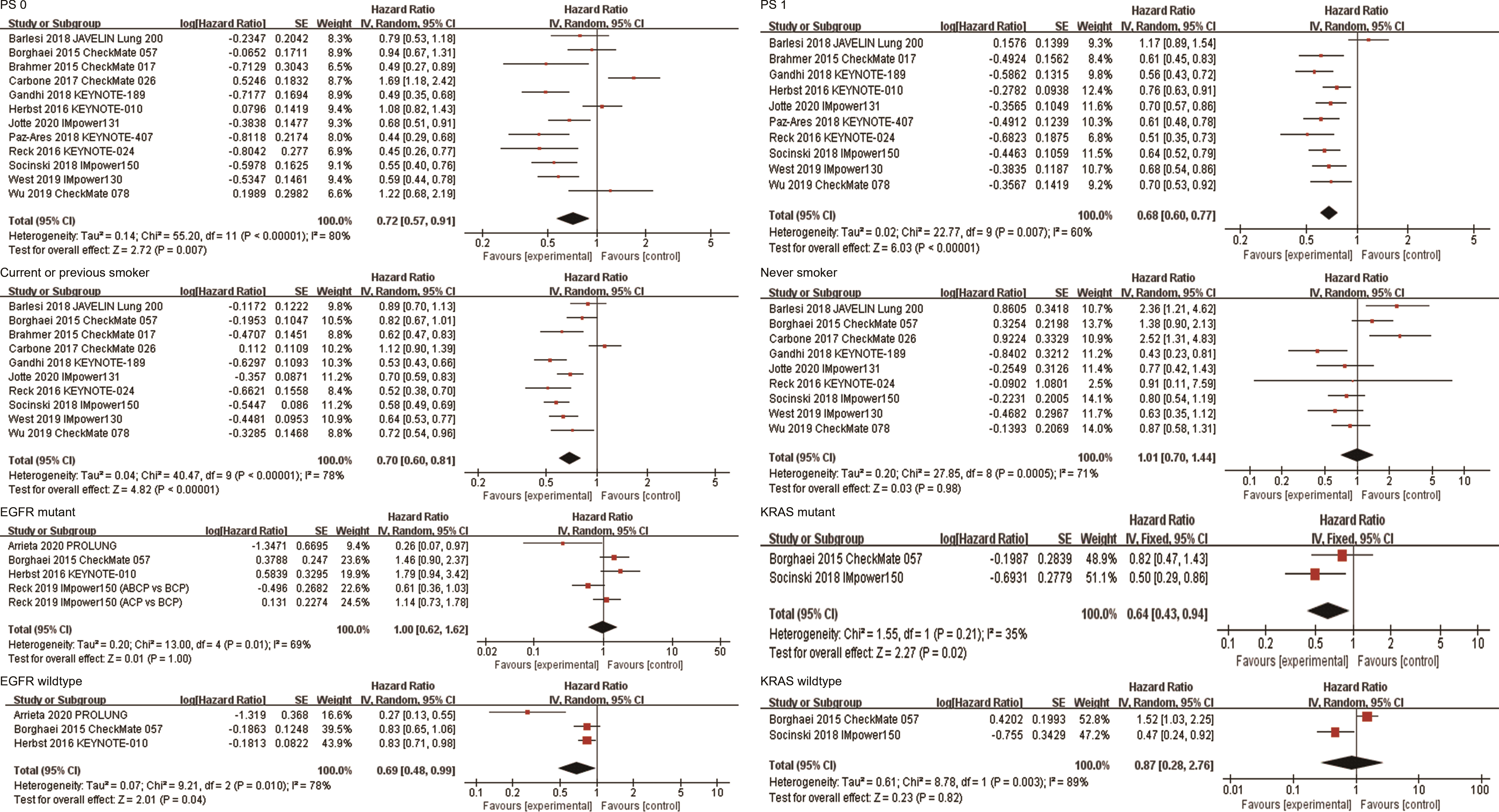

Supplement: Supplementary Figure 2 — Forest plots of hazard ratios comparing progression-free survival between patients treated with anti-PD-1/PD-L1-based therapy or non-ICI therapy according to PS score, smoking status, and driver mutations. PD-1, programmed death-1; PD-L1, programmed death-ligand 1; ICI, immune checkpoint inhibitor; PS, performance status. [file Image_2.jpeg]

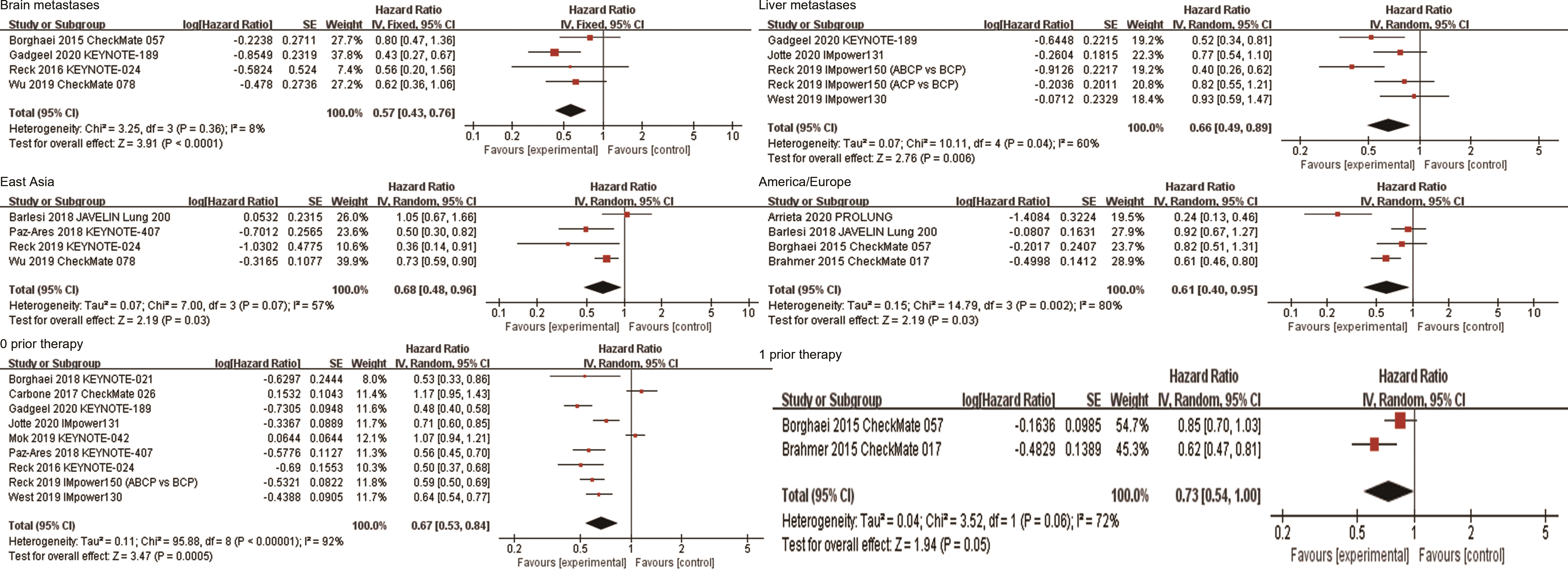

Supplement: Supplementary Figure 3 — Forest plots of hazard ratios comparing progression-free survival between patients treated with anti-PD-1/PD-L1-based therapy or non-ICI therapy according to metastatic site, region, and number of prior systemic regimens. PD-1, programmed death-1; PD-L1, programmed death-ligand 1; ICI, immune checkpoint inhibitor. [file Image_3.jpeg]

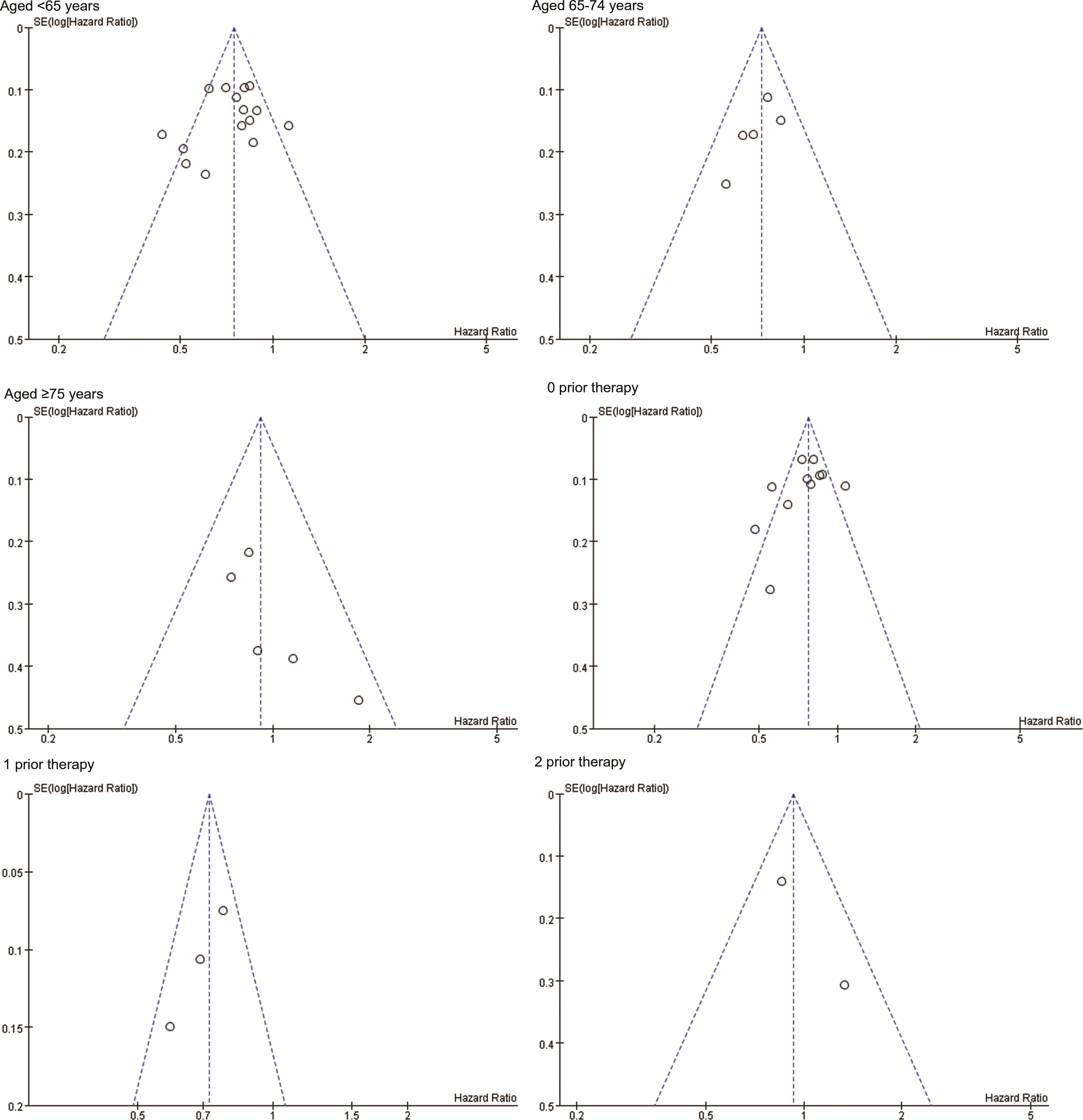

Supplement: Supplementary Figure 4 — Funnel plots of overall survival in the subgroup according to age and number of prior systemic regimens. [file Image_4.jpeg]

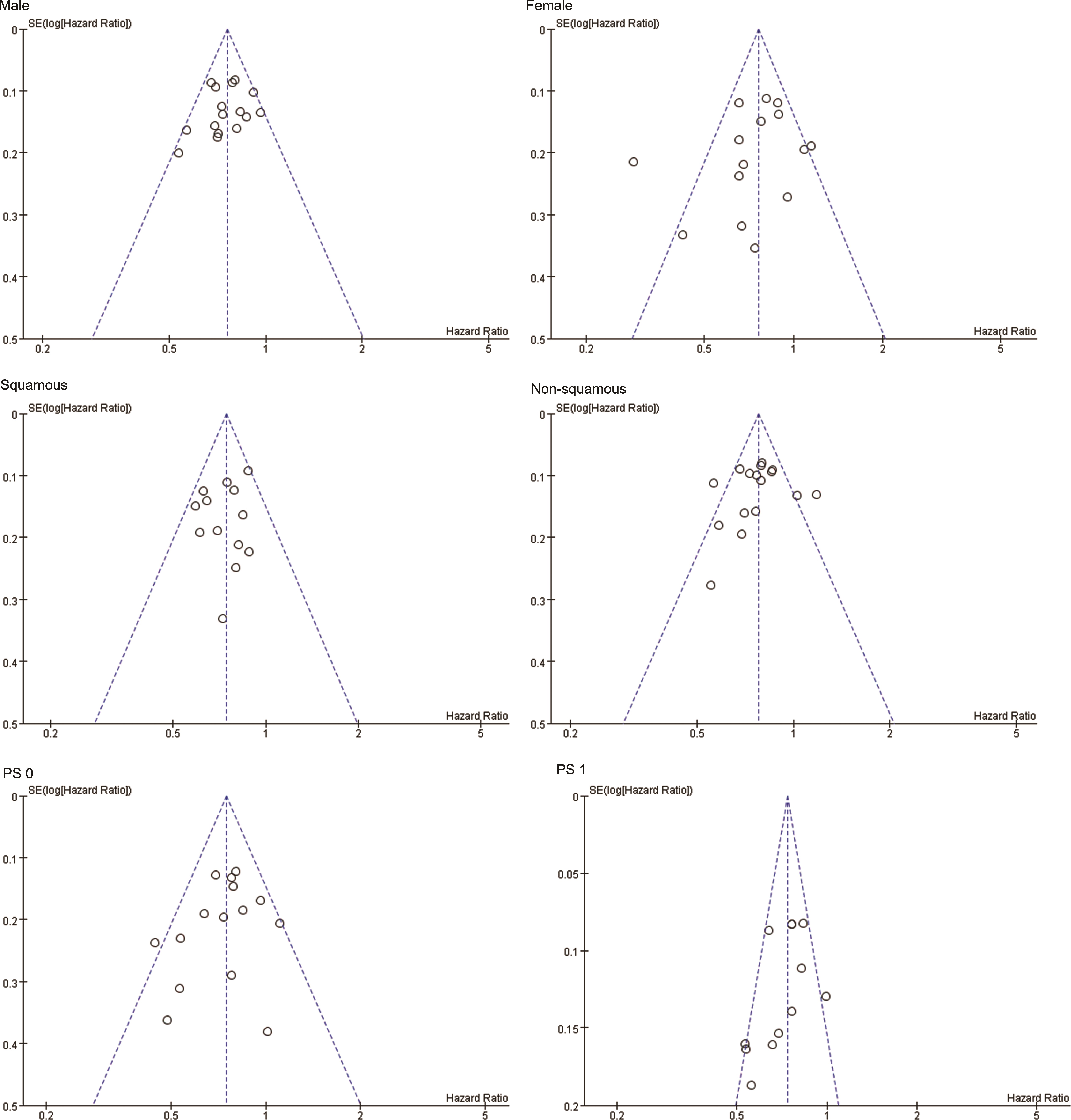

Supplement: Supplementary Figure 5 — Funnel plots of overall survival in the subgroup according to sex, histological type, and PS score. PS, performance status. [file Image_5.jpeg]

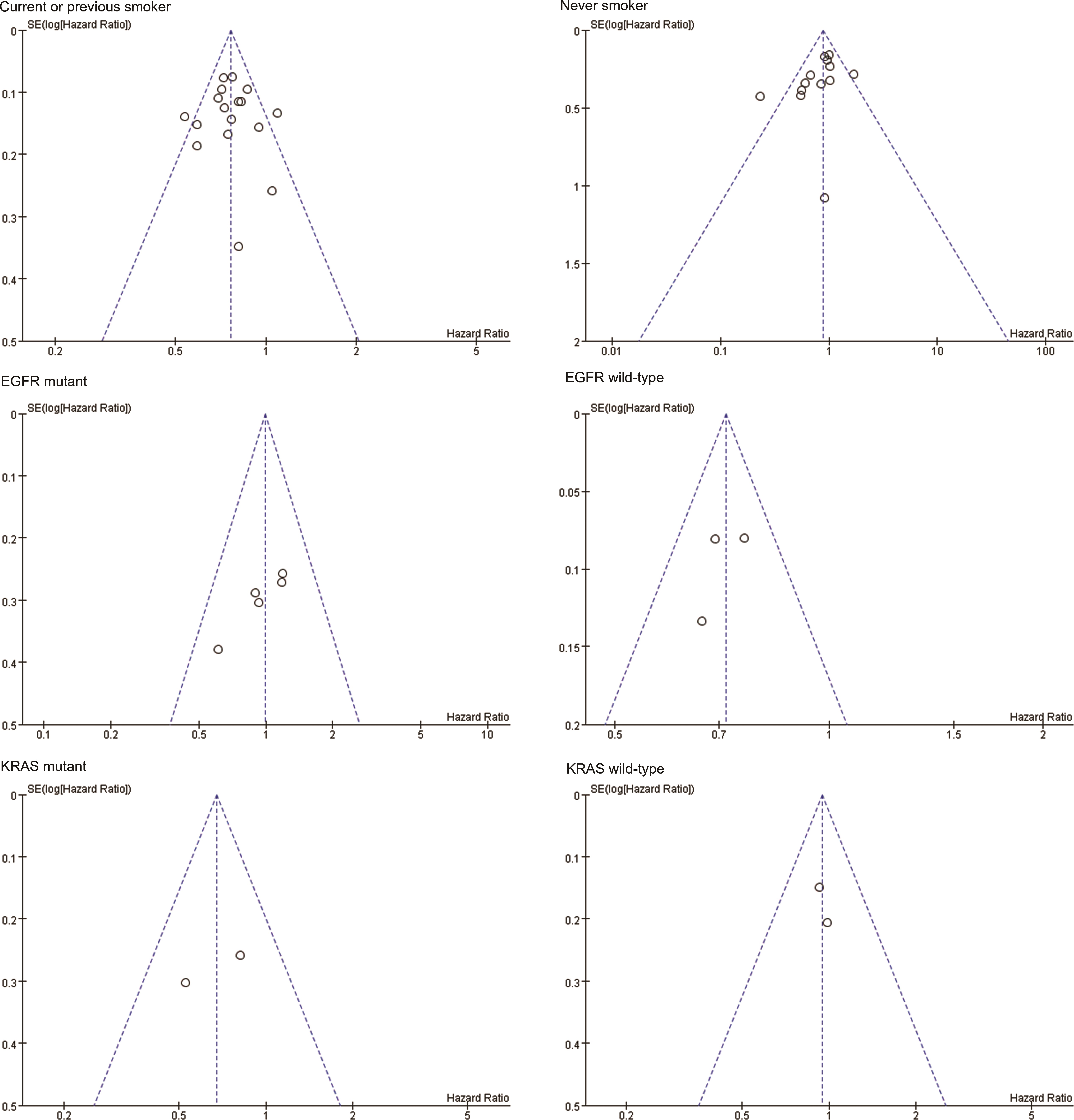

Supplement: Supplementary Figure 6 — Funnel plots of overall survival in the subgroup according to smoking status and driver mutations. [file Image_6.jpeg]

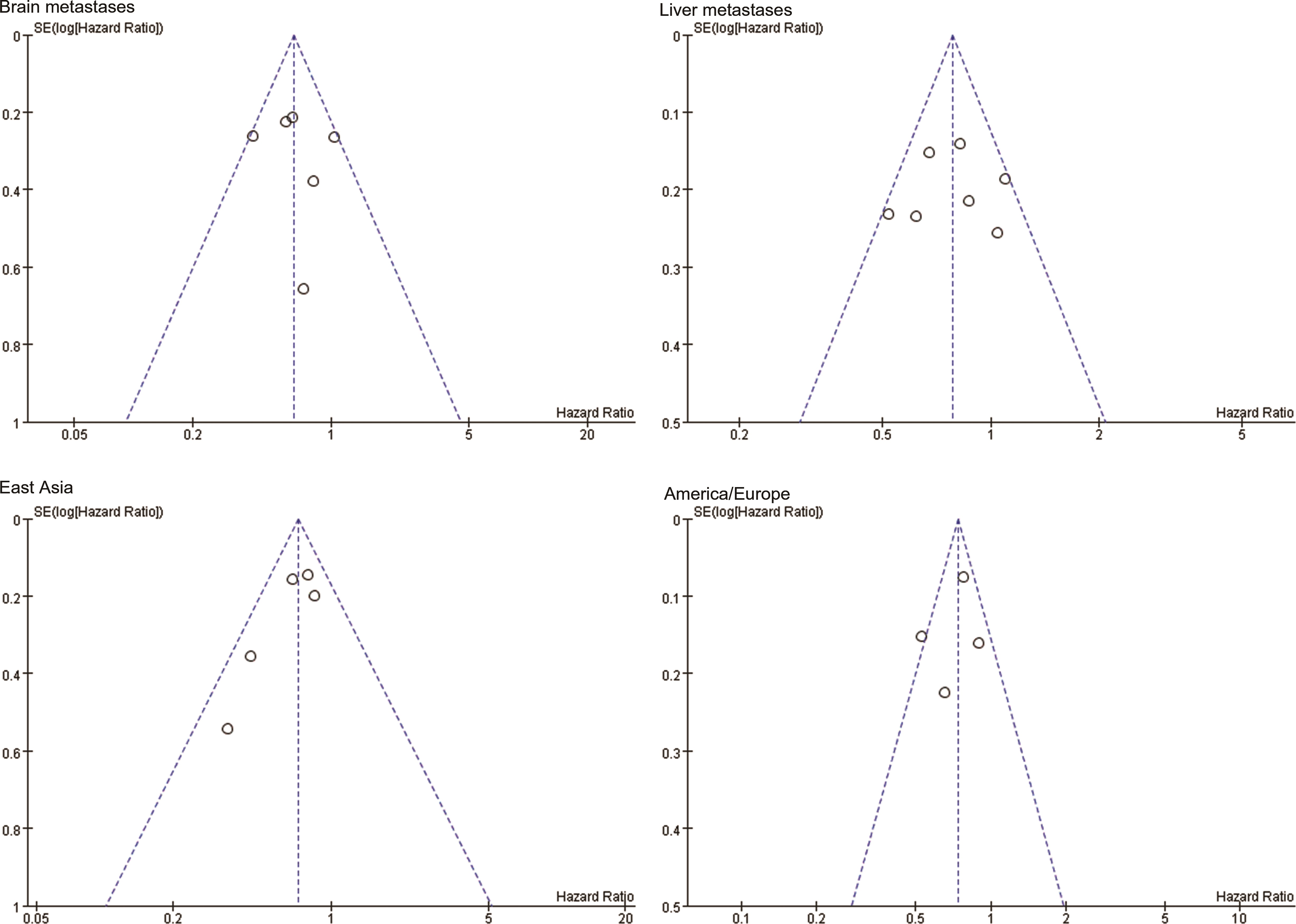

Supplement: Supplementary Figure 7 — Funnel plots of overall survival in the subgroup according to metastatic site and region. [file Image_7.jpeg]
